# Transcriptome Dynamic Analysis Reveals New Candidate Genes Associated with Resistance to Fusarium Head Blight in Two Chinese Contrasting Wheat Genotypes

**DOI:** 10.3390/ijms24044222

**Published:** 2023-02-20

**Authors:** Yunzhe Zhao, Dehua Wang, Mengqi Ji, Jichun Tian, Hanfeng Ding, Zhiying Deng

**Affiliations:** 1 Group of Wheat Quality and Molecular Breeding, State Key Laboratory of Crop Biology, Shandong Agricultural University, Tai’an 271000, China; 2 Institute of Crop Germplasm Resources, Shandong Academy of Agricultural Sciences, Jinan 250100, China

**Keywords:** wheat, *Fusarium* head blight, transcriptome, differentially expressed genes, plant defense

## Abstract

In recent years, Fusarium head blight (FHB) has developed into a global disease that seriously affects the yield and quality of wheat. Effective measures to solve this problem include exploring disease-resistant genes and breeding disease-resistant varieties. In this study, we conducted a comparative transcriptome analysis to identify the important genes that are differentially expressed in FHB medium-resistant (Nankang 1) and FHB medium-susceptible (Shannong 102) wheat varieties for various periods after *Fusarium graminearum* infection using RNA-seq technology. In total, 96,628 differentially expressed genes (DEGs) were identified, 42,767 from Shannong 102 and 53,861 from Nankang 1 (FDR < 0.05 and |log2FC| > 1). Of these, 5754 and 6841 genes were found to be shared among the three time points in Shannong 102 and Nankang 1, respectively. After inoculation for 48 h, the number of upregulated genes in Nankang 1 was significantly lower than that of Shannong 102, but at 96 h, the number of DEGs in Nankang 1 was higher than that in Shannong 102. This indicated that Shannong 102 and Nankang 1 had different defensive responses to *F. graminearum* in the early stages of infection. By comparing the DEGs, there were 2282 genes shared at the three time points between the two strains. GO and KEGG analyses of these DEGs showed that the following pathways were associated with disease resistance genes: response to stimulus pathway in GO, glutathione metabolism, phenylpropanoid biosynthesis, plant hormone signal transduction, and plant–pathogen interaction in KEGG. Among them, 16 upregulated genes were identified in the plant–pathogen interaction pathway. There were five upregulated genes, *TraesCS5A02G439700*, *TraesCS5B02G442900*, *TraesCS5B02G443300*, *TraesCS5B02G443400*, and *TraesCS5D02G446900*, with significantly higher expression levels in Nankang 1 than in Shannong 102, and these genes may have an important role in regulating the resistance of Nankang 1 to *F. graminearum* infection. The PR proteins they encode are PR protein 1-9, PR protein 1-6, PR protein 1-7, PR protein 1-7, and PR protein 1-like. In addition, the number of DEGs in Nankang 1 was higher than that in Shannong 102 on almost all chromosomes, except chromosomes 1A and 3D, but especially on chromosomes 6B, 4B, 3B, and 5A. These results indicate that gene expression and the genetic background must be considered for FHB resistance in wheat breeding.

## 1. Introduction

Since wheat (*Triticum aestivum* L.) evolved into a cultivated species approximately 10,000 years ago, it has gradually become the largest crop for human rations in the world, providing approximately 35% of the population with sufficient carbohydrates and essential nutrients. However, wheat production still faces the effects of a variety of diseases and insect pests worldwide. Of these, the occurrence of scabs is becoming increasingly serious. Since 2000, there have been 17 scab outbreaks in China [1]. In 2012, the outbreak of scab affected approximately 10 million hectares of wheat and reduced the yield by more than 2 million tons in China [2].

Fusarium head blight (FHB) is caused by infections with *Fusarium graminearum* and other types of Fusarium, which occurs during the flowering stage of wheat [3]. After infection with Fusarium, the diseased spike becomes discoloured and withered, the rachis becomes black and necrotic, and the grains shrink and become shrivelled, resulting in a significant reduction in wheat yields [1]. At the same time, the affected part accumulates a variety of toxic secondary metabolites, such as trichothecene mycotoxins and zearalenone mycotoxins [4]. Among them, deoxynivalenol is recognised as a strong carcinogenic toxin worldwide. After being consumed by humans and animals, it causes a decline in the body’s immunity and a variety of toxic reactions, which will seriously threaten human and animal health [5]. FHB brought on by *F. graminearum* was originally discovered in England in 1884 [6]. With climate warming and changes in farming systems, FHB has become a common occurrence in Asia, Europe, Oceania, and South America [7,8,9,10,11,12]. In China’s main wheat-producing areas, FHB seriously hinders the development of the wheat industry and has become an issue of great concern to wheat scientists.

Generally, FHB resistance is classified into five types, as follows: resistance to initial spike infection (type I), resistance to spike spread infection (type II), resistance to mycotoxin accumulation (type III), resistance to kernel infection (type IV), and resistance to yield reduction (type V) [13]. Of these, type II has been widely studied because of its stable resistance phenotype [3]. Wheat FHB resistance is a quantitative trait controlled by multiple genes, which are easily affected by the environment, genotype–environment interactions, and other factors. QTL/gene mapping of FHB resistance has been extensively studied, and more than 400 QTLs are distributed on all chromosomes of wheat [1]. However, there are only seven clear resistance genes known, namely *Fhb1* to *Fhb7*; therefore, new genes/QTLs and new varieties with FHB resistance still need to be studied.

Transcriptome sequencing (RNA-Seq) technology has rapidly developed in recent years. This technology can quickly and comprehensively obtain the complete expression information of a sample in a certain state through high-throughput sequencing of the transcriptome product mRNA. It has been widely used in all aspects of plant research, especially abiotic and biotic stresses. A few studies have reported on wheat FHB resistance using this method. Transcriptome analysis showed that protein serine/threonine kinase and LRR-RK are associated with susceptibility to FHB, whereas several ethylene-responsive, *WRKY*, *Myb*, *bZIP*, and *NAC*-domains containing transcription factors were also found to be associated with susceptibility [14]. Li et al. [15] conducted a comparative transcriptome analysis to identify genes that are differentially expressed in FHB-resistant and FHB-susceptible wheat lines grown under field conditions for various periods after *F. graminearum* infection and determined the chromosomal distribution of the differentially expressed genes (DEGs). XU et al. [16] identified the FHB resistance genes of wheat variety Xinong 979 by transcriptome sequencing and screened detoxification-related protein genes, such as UDP-glucosyltransferase genes. Su et al. [17] used transcriptomics and metabolomics to track and analyse the infection process of *F. graminearum* and found that a large number of secondary metabolites, such as flavonoids, plant hormones, tryptamines, phenolic amines, and alkaloids, were involved in wheat scab resistance.

Although a few studies have been conducted on FHB resistance using transcriptome sequencing, the disease resistance mechanism is still not very clear because of the huge wheat genome and the complicated traits of FHB resistance. Therefore, further studies are required. Moreover, most previous studies used materials with high resistance and high sensitivity because of their ease of study, few studies used medium-sensitivity (MS) and medium-resistance (MR) materials. In fact, there are few varieties with high and medium resistance in production, most of which are highly susceptible and moderately susceptible. Therefore, the mechanism of resistance should be further studied. Meanwhile, the release of the complete wheat genome sequence and detailed annotations allows for the exploratory analysis of DEGs, specifically in known FHB resistance QTL/gene regions. Therefore, to explore the molecular differences in FHB resistance between MS and MR wheat strains, two special genotypes, Shannong 102 (MS) and Nankang 1 (MR), were inoculated with *F. graminearum*. The inoculated spikelets were analysed at 0, 48, and 96 h using RNA sequencing technology. The results will provide insight into the genetic basis of moderate FHB resistance and suggest a molecular strategy for breeding wheat cultivars with this trait. Furthermore, this study provides new potential genes for breeding applications.

## 2. Results

### 2.1. Quality Assessment of Transcriptome Sequencing

**Figure 1:**
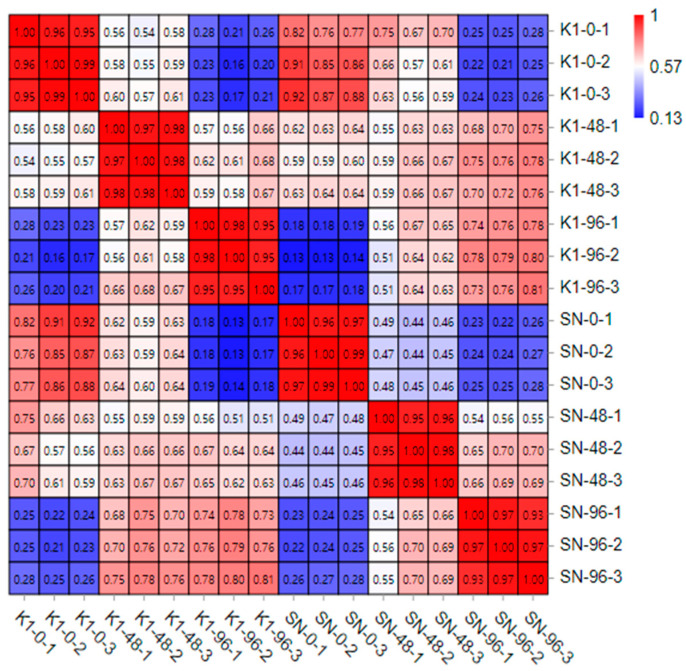
Statistical correlation coefficients of biological replicates used to assemble sequences (*p* < 0.05). The SN in the sample indicates wheat Shannong 102, and K1 indicates wheat Nankang 1. The number 0 indicates the sample before inoculation, and 48 and 96 indicate the time (h) after inoculation. Figure 1, Figure 2 and Figure 3 indicate the three replicates. The same goes for subsequent figures and tables.

According to the analysis of the sample correlation thermogram (Figure 1), the repeatability of the samples in the group was good, and the correlation coefficients were all above 0.88. The correlation distances of samples under different treatment conditions were large, which showed that the samples were obviously affected by the experimental treatment. By comparing the sequence data between Shannong 102 and Nankang 1 in the wheat genome IWGSC_RefSeq_v1.1 (https://wheat-urgi.versailles.inra.fr/Seq-Repository/Annotations; accessed on 26 March 2022) and filtering the low-quality data, 1,009,677,180 total reads were obtained. The number of reads in the unique mapped reference genome accounted for 76.02–91.05% of the effective reads, and the total number of mapped reads in the reference genome accounted for 79.47–96.06%. The percentage of Q30 bases was more than 93.66%, and the GC content ranged from 58.50 to 61.37% (Table 1). From these data, the sequencing quality was high and met the requirements for subsequent analysis.

### 2.2. Analysis of DEGs

**Figure 2:**
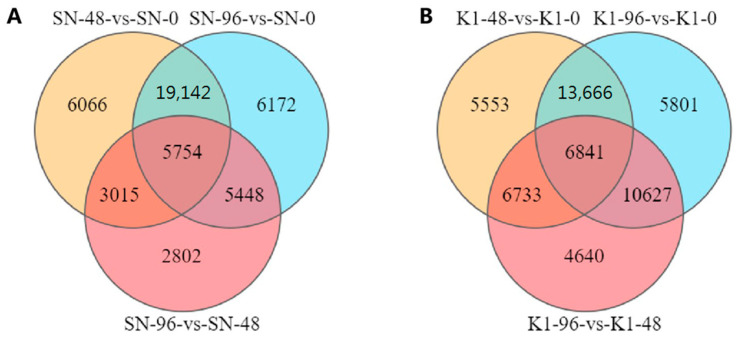
Venn plot of differentially expressed genes in the three stages. (**A**) Shannong 102; (**B**) Nankang 1.

Using an FDR < 0.05 and |log2FC| > 1 as screening criteria, 96628 total DEGs were identified. Of these, there were 42767 DEGs in Shannong 102 and 53861 DEGs in Nankang 1. Overall, the number of DEGs in Shannong 102 was slightly lower than that in Nankang 1 (Figure 2).

**Figure 3:**
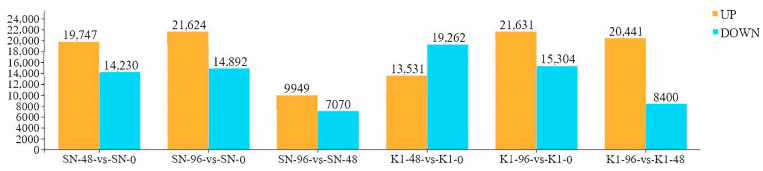
Statistical analysis of differentially expressed genes.

In Shannong 102, 42,767 DEGs were identified. Of these, 5754 were found to be shared among the three time points (Figure 2A). By comparing SN-48 vs. SN-0, SN-96 vs. SN-0, and SN-96 vs. SN-48 groups, the numbers of upregulated DEGs were 19,747, 21,624, and 9949, respectively, and the numbers of downregulated DEGs were 14,230, 14,892, and 7070, respectively (Figure 3).

**Figure 4:**
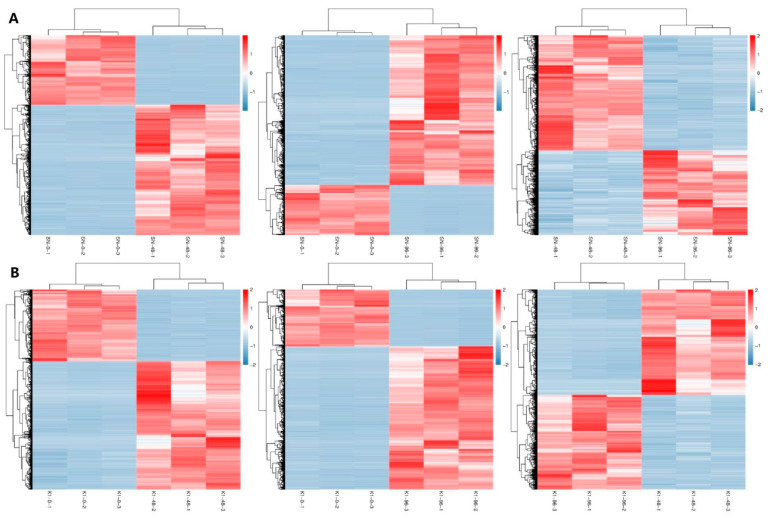
Expression pattern clustering of differentially expressed genes (DEGs). (**A**) Shannong 102; (**B**) Nankang 1.

However, in Nankang 1, 53,861 total DEGs were found. Of these, 6841 DEGs were shared among the three time points (Figure 2B). Pairwise comparisons among K1-0, K1-48, and K1-96 indicated 13,531, 21,631, and 20,441 upregulated DEGs in the K1-48 vs. K1-0, K1-96 vs. K1-0, and K1-96 vs. K1-48 comparisons, respectively (Figure 3), and there were 19,262, 15,304, and 8400 downregulated genes, respectively (Figure 3). The expression pattern clustering of DEGs indicated that the expression of most genes was affected by the different treatments (Figure 4).

### 2.3. GO Analysis of DEGs

**Figure 5:**
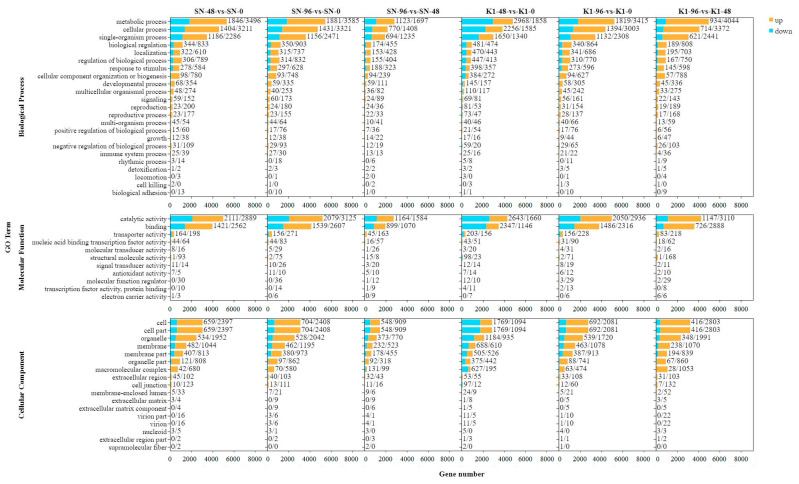
Gene Ontology classification of differentially expressed genes (DEGs).

GO analysis revealed 23, 11, and 17 functional classifications in biological process (BP), molecular function (MF), and cellular component (CC) categories, respectively, in Shannong 102. In the BP classification, most DEGs were enriched in the metabolic processes, cellular processes, and single-organism processes. In the MF classification, the most abundant DEGs were related to catalytic activity and binding, and in the CC classification, the majority of DEGs were enriched in the cell, cell part, and organelle (Figure 5). The was similar to the results of the GO analysis for Nankang 1.

The enrichment of upregulated genes in Shannong 102 and Nankang 1 at 0, 48, and 96 h after inoculation (Tables S2 and S3) was mainly distributed in metabolic processes, cellular processes, and single-organism processes. However, in the MF classification, the upregulated DEGs were mainly enriched in seven subclasses, such as catalytic activity, binding, and transporter activity. In the CC classification, most of the upregulated genes were enriched in the cells, organelles, and membranes. The enrichment of downregulated DEGs in Shannong 102 and Nankang 1 are shown in Tables S4 and S5. By comparing the DEGs between Shannong 102 and Nankang 1 (Figure 3), Shannong 102 had more upregulated genes than Nankang 1 at 48 h after inoculation, but Nankang 1 had more upregulated genes than Shannong 102 at 96 h after inoculation.

### 2.4. KEGG Analysis of DEGs

In total, 25,244 genes with pathway annotations were obtained by comparing the DEGs between Shannong 102 and Nankang 1 in the KEGG database. For both Nankang 1 and Shannong 102, after *F. graminearum* inoculation, the numbers of DEGs enriched in metabolic pathways (ko01110) and biosynthesis of secondary metabolites (ko01100) were significantly higher than those in other pathways (Table 2 and Table 3 and Figure 1), which was consistent with the metabolic process enrichment results of the GO analysis.

Taking a q value < 0.05 as the screening condition, we analysed the metabolic pathways of upregulated and downregulated DEGs in Shannong 102 and Nankang 1 at different time points after inoculation (Tables S6 and S7). The results showed that the pathways associated with upregulated DEGs were mainly related to disease resistance, such as phenylpropanoid biosynthesis (ko00940), plant–pathogen interaction (ko04626), plant hormone signal transduction (ko04075), glutathione metabolism (ko00480), and MAPK signalling pathway plant (ko04016). With an extension of the inoculation time, the number of upregulated genes in disease resistance-related pathways decreased. Downregulated genes were mainly enriched in the biosynthesis of secondary metabolites (ko01110), carbon metabolism (ko01200), and starch and sucrose metabolism (ko00500). Therefore, the FHB resistance of these two genotypes can be improved by upregulating the expression of genes related to glutathione metabolism, phenylpropane biosynthesis, MAPK signalling pathway plant, and plant–pathogen interaction. The differences in DEG expression levels between Shannong 102 and Nankang 1 might lead to differences in disease resistance between the two samples. This will be verified by RT-qPCR results.

### 2.5. Comparing DEGs between Shannong 102 and Nankang 1 after Inoculation

**Figure 6:**
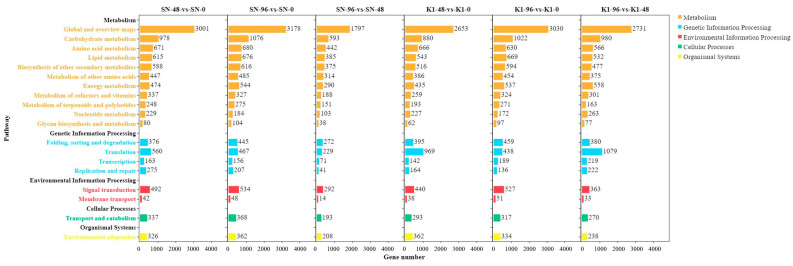
KEGG annotation functional enrichment of pathways.

**Figure 7:**
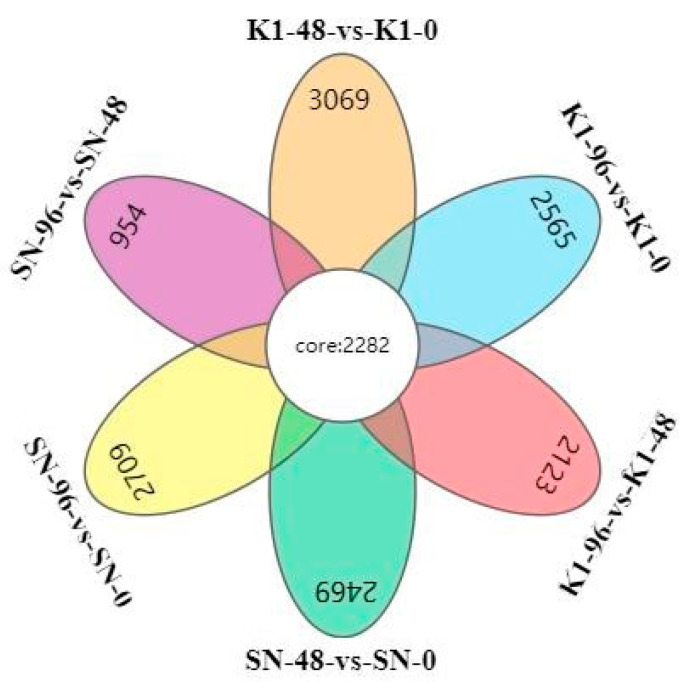
Comparison of differentially expressed genes at three stages in Shannong 102 and Nankang 1.

**Figure 8:**
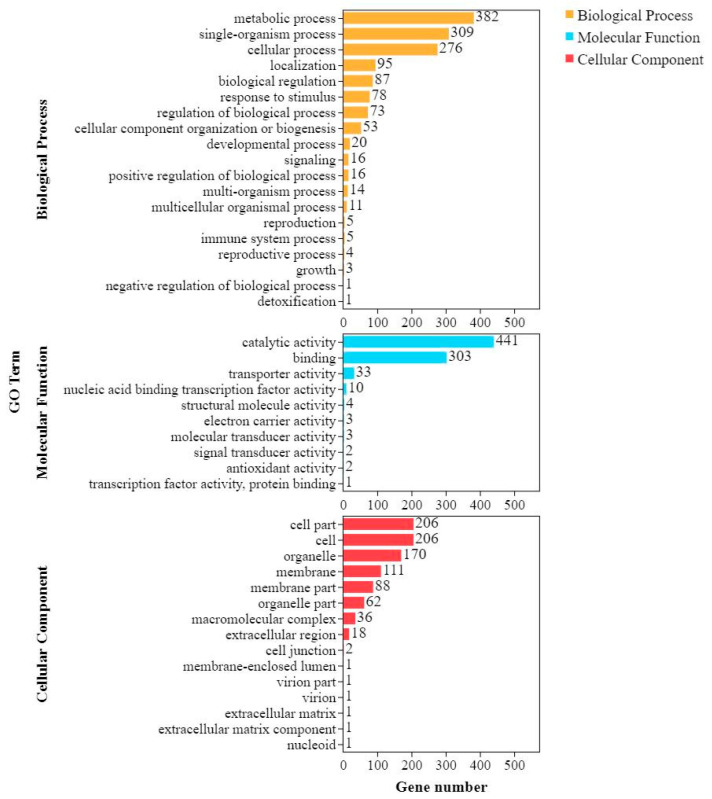
Gene Ontology classification of shared differentially expressed genes (DEGs) in Shannong 102 and Nankang 1.

**Figure 9:**
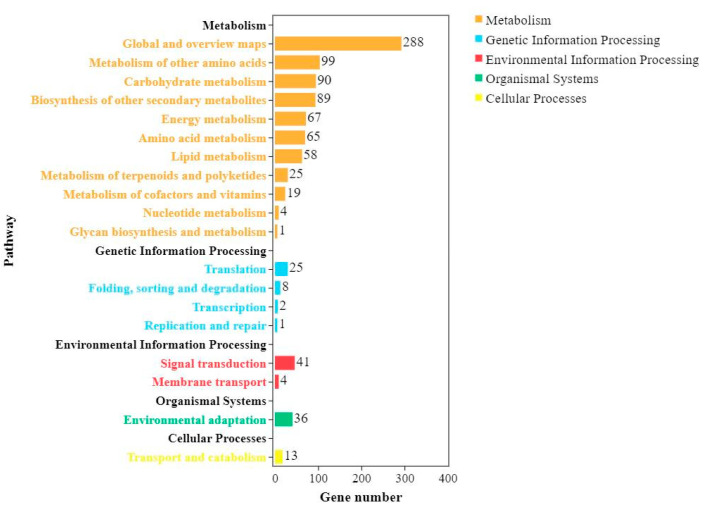
KEGG classification of shared differentially expressed genes (DEGs) in Shannong 102 and Nankang 1.

After inoculation for 48 h, the number of upregulated genes in Nankang 1 was significantly lower than that of Shannong 102, but at 96 h, the number of DEGs in Nankang 1 was higher than that in Shannong 102 (Figure 3). GO and KEGG analyses also proved this (Figure 5 and Figure 6). These results indicated that Shannong 102 and Nankang 1 had different defensive responses to *F. graminearum* in the early stages of infection. To further dissect the differences between the two materials, we compared the DEGs, and in total, 2282 genes were differentially expressed at three time points (Figure 7). The GO and KEGG analyses of these DEGs showed that the following pathways were involved in disease resistance: response to stimulus pathway in GO, glutathione metabolism, phenylpropanoid biosynthesis, plant hormone signal transduction, and plant–pathogen interaction in KEGG (Figure 8 and Figure 9, and Table 4).

### 2.6. DEGs Encoding Pathogenesis-Related Proteins

**Figure 10:**
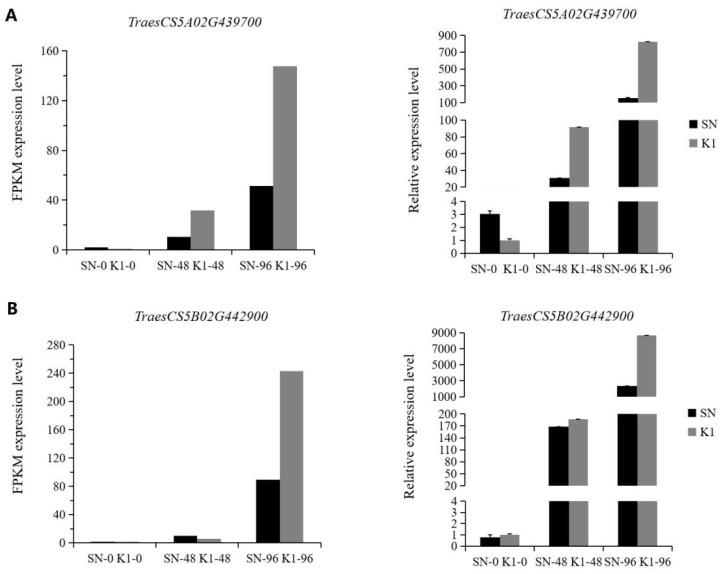

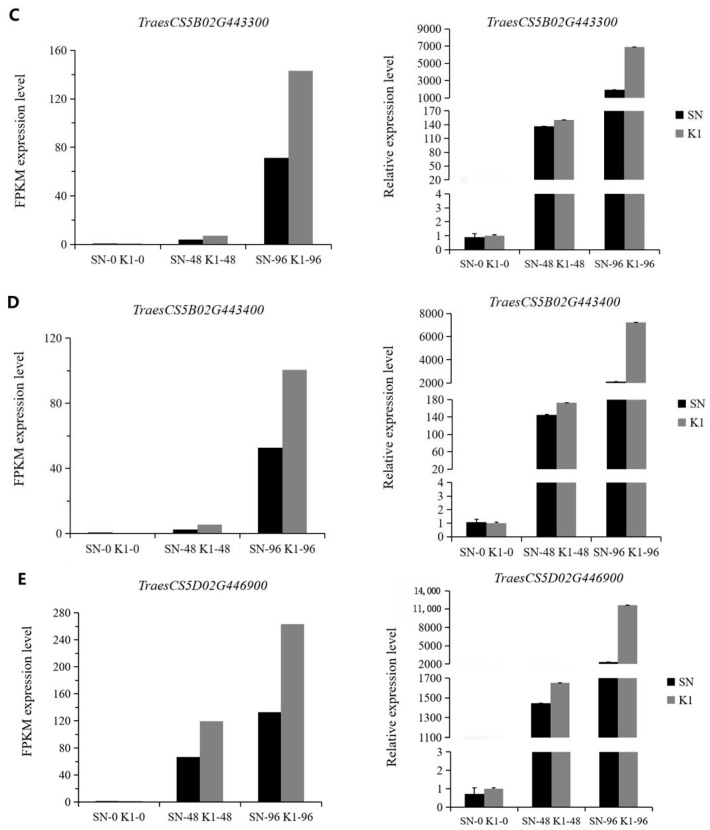
Comparison of RNA-seq and RT-qPCR data for selected genes. (**A**) Differences in expression levels of *TraesCS5A02G439700*, (**B**) Differences in expression levels of *TraesCS5B02G442900*, (**C**) Differences in expression levels of *TraesCS5B02G443300*, (**D**) Differences in expression levels of *TraesCS5B02G443400*, (**E**) Differences in expression levels of *TraesCS5D02G446900*.

After screening genes with an FDR < 0.05 and |log_2_FC| > 1, 16 total upregulated genes were found in the plant–pathogen interaction pathway (14 PRMS genes and 2 RPS2 genes; Table S8). Of these, there were five upregulated genes, TraesCS5A02G439700, TraesCS5B02G442900, TraesCS5B02G443300, TraesCS5B02G443400, and TraesCS5D02G446900, which had significantly higher expression levels in Nankang 1 than in Shannong 102. The PR proteins they encode are PR protein 1-9, PR protein 1-6, PR protein 1-7, PR protein 1-7, and PR protein 1-like. The relative expression levels of the five DEGs in the two strains were then analysed by RT-qPCR to verify the reliability of the transcriptome data. The results showed that the transcriptome sequencing results of the five genes were consistent with the quantitative fluorescence results (Figure 10). An early disease resistance phenotype identification revealed that Nankang 1 (MR) had better disease resistance than Shannong 102 (MS), indicating that these five genes might play an important role in regulating the resistance of plants to *F. graminearum* infection.

### 2.7. Distribution of DEGs on Chromosomes

The number of DEGs in Nankang 1 was higher than that in Shannong 102 on almost all chromosomes, except chromosomes 1A and 3D. In addition, using the special molecular markers, it was found that Nankang 1 had the Fhb1, Fhb4, and Fhb5 QTL/gene regions, whereas Shannong 102 had the Fhb2 and Fhb4 QTL/gene regions (Table S1). Because the two experimental strains contained known disease resistance genes, we focused on the DEGs on the chromosomes of known disease resistance genes. Shannong 102 had 208 and 205 DEGs on 6B and 4B, and Nankang 1 had 360, 268, and 327 on 3B, 4B, and 5A, respectively. On chromosome 4B, we found that 81 genes were differentially expressed between the two strains. Meanwhile, the number of DEGs on these five chromosomes in Nankang 1 was higher than that in Shannong 102. These could be beneficial for resistance in Nankang 1 (Table S9).

## 3. Discussion

Facing the increasingly serious epidemic of wheat scab, the scab-resistant varieties have become the focus of wheat breeding research. Wheat scab resistance is a quantitative trait controlled by multiple genes, and there are some major disease resistance genes [13]. Using different types of genetic mapping techniques, the main QTL/genes, including *Fhb1* to *Fhb7*, associated with resistance to scab, have been finely mapped, and a series of molecular markers has been developed for assisted selection breeding [18,19,20,21,22,23,24,25]. Using marker-assisted backcross, Ma et al. [1] transferred *Fhb1*, *Fhb2*, *Fhb4*, and *Fhb5* from Wangshuibai to 40 excellent varieties introduced from different wheat-producing areas and cultivated more than 70 FHB-resistant lines with different QTL combinations. Compared with their recurrent parents, the lines carrying *Fhb4* and/or *Fhb5* showed significantly better type I resistance, and the lines carrying *Fhb1* and/or *Fhb2* showed significantly better type II resistance. The breeding lines carrying these four genes showed significant improvements in resistance, which was equivalent to that of Wangshuibai and Sumai 3 [1]. Four FHB resistance genes, *Fhb1*, *Fhb2*, *Fhb4*, and *Fhb5*, were polymerised into the same susceptible variety, Aikang 58, and the disease resistance of some lines was close to that of Sumai 3 and Wangshuibai [26]. Molecular marker detection showed that Shannong 102 contained *Fhb2* and *Fhb4,* and Nankang 1 contained *Fhb1*, *Fhb4*, and *Fhb5* in our study. Some lines from the recombinant inbred line (RIL) population derived from Shannong 102 and Nankang 1 showed high resistance, and the type II resistance was close to that of Sumai 3 and Wangshuibai. Elite lines with high resistance to scabs from the RIL population were identified (unpublished data). These results indicate that the gene aggregation of FHB resistance can improve FHB resistance, but the mechanism by which the genes interact to increase resistance remains unclear.

At present, most popularised cultivated varieties are moderately or highly susceptible to scabs. Highly resistant varieties, such as Sumai 3 and Wangshuibai, have failed to be popularised in a large area because of their poor agronomic characteristics. Although the agronomic characteristics of Yangmai 158 and other moderately resistant varieties are good, disease is still inevitable in years of severe FHB epidemics. Among them, Shannong 102 was selected for this experiment, with good agronomic characteristics and medium sensitivity to scab. The average yield per hectare in a 3-year regional experiment was 8316 kg. The genetic population constructed by crossing Shannong 102 and Nankang 1, with medium resistance to scab, combined with molecular marker-assisted selection and agronomic characters, can obtain lines with excellent resistance and good agronomic characteristics. Therefore, the combination of molecular marker-assisted breeding and traditional breeding will play an increasingly important role in wheat breeding in the future [2]. At present, gratifying progress has been made in the improvement of wheat scab resistance, but the final solution to this problem is limited because its resistance mechanism is not clear [1]. In the future, we should not only increase the breeding of scab resistant varieties, but also strengthen research on the resistance mechanism to provide a theoretical basis for the improvement of wheat scab resistance [2].

Wheat is allohexaploid and contains three genomes, A, B, and D. The genome is huge (three ancestral genomes, approximately 17,000 Mbp [27]. Therefore, it is not easy to carry out genetic research on complex traits, which also greatly hinders the research on FHB resistance mechanisms. In recent years, RNA-seq technology based on the Illumina sequencing platform has become a powerful tool for the rapid and comprehensive establishment of plant basic molecular platforms [28,29] and also provides convenience for exploring the mechanism of wheat scab resistance [30,31]. In this study, the transcriptomes of Shannong 102 and Nankang 1 at 0, 48, and 96 h after inoculation were sequenced, and the gene expression in response to scab was obtained.

Plant disease resistance is a fine molecular process regulated by a gene network that involves cellular and molecular events and signalling pathways. Functional annotation of the DEGs was performed. The most common classifications are the defence-related plant hormone synthesis and pathways [32,33], phenylpropionic acid synthesis pathway [34], production and clearance of reactive oxygen species, synthesis of antibacterial compounds [35], detoxification, and cell wall reinforcement [36], among others. After infection, many of these pathway products were upregulated or inhibited, indicating that they are involved in the defense response. The pathways of the upregulated genes of Shannong 102 and Nankang 1 were consistent. BPs included biological regulation, response to stimulus, immune system processes, and the positive regulation of biological processes. The classification of CCs included cells, organelles, and membranes, and the MFs included catalytic activity, binding, and transporter activity. Some of these were similar to those in previous research.

The pathways involved in the KEGG analysis of differential expression might be related to the resistance of Shannong 102 and Nankang 1 to scab, and both contained two highly enriched pathways, that is, biosynthesis of secondary metabolites (ko01110) and metabolic pathways (ko01100). This provides a reference for research on the FHB resistance mechanism of Shannong 102 and Nankang 1. A previous study showed that 70 pathways are involved in metabolism after *F. graminearum* infection using Xinong 979, based on transcriptome analysis [8]. The first four main pathways are the protein processing pathways in the endoplasmic reticulum, photosynthesis-antenna protein pathway, starch and sucrose metabolism pathway, and plant hormone signal transduction pathway [16]. This is similar to our results. However, the differences in the first few pathways with the highest enrichment led to differences in the FHB resistance mechanisms between Nankang 1 and Xinong 979.

Some different pathways that accumulated in one of the two genotypes were upregulated or downregulated, which is more likely to be related to disease resistance or susceptibility [1]. Therefore, in the transcriptome analysis of Shannong 102 and Nankang 1, we focused on the differences in DEGs between the two strains and found that Nankang 1 showed alterations in glutathione metabolism (ko00480), phenylpropanoid biosynthesis (ko00940), plant–pathogen interaction (ko04626), plant hormone signal transduction (ko04075), and other pathways, with respect to the number of upregulated genes. Transcription products, proteins, and metabolites related to the phenylpropanoid pathway, such as phenylalanine ammonia lyase (PAL) and hydroxycinnamic acid amides, are only induced in disease-resistant strains or induced with higher abundance. Gunnaiah and Kushalappa [37] showed that the phenylpropanols related to disease resistance that accumulate in Sumai 3 mainly consist of syringyl-rich monomeric alcohols and their glycosides, which are lignin and antibacterial plant antitoxins. Biosynthetic precursors, therefore, might play an important role in inhibiting the penetration and growth of fungi. In addition, because SA can be synthesised through the PAL pathway to respond to pathogen attacks, the phenylpropanoid pathway is related to the SA signalling pathway.

A pathogenesis-related protein refers to a type of water-soluble protein produced by plants after infection by pathogens or stimulation with non-biological factors. In recent years, researchers have discovered that PR protein genes also play an important role in the disease resistance of crops. In a study of FHB, glucanase and chitinase were found to play an important role in the resistance and defense response of wheat to FHB, and they can play a direct role in the “damage of fungal structural barrier” or indirectly through the “activity of fungal cell wall damage products” [38]. Li et al. [39] used the resistant variety Sumai 3 and susceptible variety Y1193-6 as experimental materials to study their difference of FHB resistance and found that some PR genes of the two materials were upregulated after inoculation.

In this study, many PR protein genes were differentially upregulated, and these were enriched in the plant–pathogen interaction (ko04626) pathway. The gene expression showed an increasing trend with the inoculation time, indicating that PR protein genes play an important role in regulating plant disease resistance after *F. graminearum* infection. The expression levels of PR-1 and PR-2 (β-1,3-glucanase), PR-3 (chitinase), PR-4 (he-vein-like protein), and PR-5 (thaumatin-like protein) peaked at 36 to 48 h using Sumai 3 and Wheaton as experimental materials. However, there was no significant difference in the expression of PR protein genes between Sumai 3 and Wheaton [40]. However, in this study, it was found that most of the disease course-related proteins or disease resistance genes screened from the two wheat materials, Nankang 1 and Shannong 102, were upregulated and differentially expressed between the two strains, and there was a large difference in the gene expression between these two genotypes, which was not consistent with the results of Pritsch et al. [40]. To verify the accuracy of the transcriptome data, we selected five PRMS (pathogenesis-related protein 1)-related genes in the plant–pathogen interaction (ko04626) pathway for RT-qPCR analysis. The results of this were consistent with the transcriptome results, that is, the upregulated or downregulated gene expression trend was the same between them. This indicated that the transcriptome test results were more reliable. Pan et al. [14] found that all PR1, PR1-1, and PR-4 genes were upregulated by *F. graminearum*, but none of them were expressed at higher levels in any resistant genotype, relative to levels in the susceptible Shaw strain. However, in our study, PR-related genes were also upregulated, but the expression levels were different between the two genotypes, which was different from the previous results [14]. This could be caused by the difference in strains with different known FHB QTLs/genes or gene interactions. PR genes can be expressed, or not, which might be closely related to the genetic background of the plant lines. In addition to finding that PRM-related genes are closely related to wheat disease resistance, the overexpression of RPS2 (disease resistance protein RPS2) and RPM1 (disease resistance protein RPM1) were found to provide broad-spectrum disease resistance in rice [41]. In this study, it was found that two RPS2 protein gene upregulation events were identified in the plant–pathogen interaction pathway, indicating that they might be related to resisting invasion by *F. graminearum* and play a positive regulatory role in this process. However, the specific mechanism needs to be further studied in the future.

## 4. Materials and Methods

### 4.1. Plant Materials

Two wheat (*T. aestivum* L.) genotypes were used in this study, Shannong 102 (a cultivar with MS to FHB) and Nankang 1 (a line with MR to FHB). Seeds were kindly provided by Dr. Jichun Tian (Shandong Agricultural University). After screening two genotypes using the special molecular markers for Fhb1, Fhb2, Fhb4, and Fhb5 (Figure S1), it was found that Nankang 1 had the QTL/gene regions of Fhb1, Fhb4, and Fhb5, whereas Shannong 102 had the QTL/gene regions of Fhb2 and Fhb4 (Table S1).

The seeds of the two genotypes were germinated and vernalised for an additional 4 weeks (4 °C, 12 h light/dark regime) before being transferred to the greenhouse. Plants were potted in a mixture of compost, sand chalk, and common soil. Each sample was planted in an individual pot (30 cm in diameter and 25 cm in depth, with three seedlings) and with 10 replicates (pots). The temperature of the greenhouse was gradually increased from 15 °C/13 °C during the day/night to 20 °C/18 °C, and a 16 h/day photoperiod was used at the time of anthesis.

In this study, mixed conidiospore suspensions of 7136, F301, F609, and F15 virulent strains of *F. graminearum* were obtained from the courtesy of Nanjing Agricultural University. The pathogen was inoculated in mung bean medium and vortexed at 150 rpm at 25 °C for 4–5 days. After culturing and filtering, the mass of conidia was examined under a microscope. Then, the four pathogen strains were mixed equally and stored at 4 °C for later use. Wheat was inoculated with 20 μL of the *F. graminearum* conidia suspension (4 × 105 spores/mL), applied to a pair of florets in the middle of the spike (or 1/2 position of the spike) during flowering. The entire wheat spike was then covered with a self-sealing bag to retain moisture and sprayed with water 1–2 times per day, and the self-sealing bag was removed after 3 days.

### 4.2. Sample Preparation for RNA-Seq Analysis

The entire spikes of the two genotypes were collected at 0 h (inoculated with fresh sterile water), 48 h, and 96 h after inoculation with three biological replicates, and these were named SN-0, SN-48, and SN-96 for Shannong 102 and K1-0, K1-48, and K1-96 for Nankang 1. In total, 18 samples of the two genotypes were collected.

### 4.3. RNA Extraction and Sequencing Analysis

Total RNA was extracted using TRIzol reagent (Life Technologies, Carlsbad, CA, USA), followed by treatment with DNase I (Ambion, Austin, TX, USA), according to the manufacturer’s protocol.

The Illumina Novaseq 6000 (Illumina, Inc. 9885 Towne Centre Drive, San Diego, CA, USA) was used to sequence the cDNA library. Single-end sequencing ensured that the sequencing depth of each sample exceeded 10 Gb. RNA-Seq was performed by Gene Denovo Co. Ltd. (Guangzhou, China).

### 4.4. RNA-Seq Data Analysis and Bioinformatics Analysis

FASTP (version 0.18.0) [42] was used to filter the raw data to obtain clean data. The BWA software (version 0.7.12) [43] was used with the mem algorithm to compare the filtered reads to the Chinese spring wheat reference genome (IWGSC_RefSeq_v1.1). After the comparison, the results were marked with the software Picard (version 1.129), as well as statistical marking of the depth and coverage of the reads.

### 4.5. Differential Expression Gene Analysis and Gene Functional Annotation

The fragments per kilobase of transcript per million fragments mapped value [44] was used to reflect the gene expression and analyse the differential genes. The differential expression of genes between the two groups was analysed using the DESeq2 (version 1.30.0). A false detection rate (FDR) < 0.05, and |log2fold-change (FC)| > 1 were used as the screening criteria to obtain the DEGs between the two samples.

The DEG sequences were compared with the Gene Ontology (GO) database (http://www.geneontology.org/; accessed on 28 March 2022), and the function was annotated. The DEGs were also compared with the Kyoto Encyclopedia of Genes and Genes (KEGG) database using blastx, and the corresponding pathway annotation information was obtained.

### 4.6. Real-Time Quantitative PCR (RT-qPCR) Analysis

The DEGs in pathways related to plant disease resistance and immunity were selected for real-time fluorescent quantitative PCR verification (Table 5). Actin (https://www.ncbi.nlm.nih.gov/nuccore/AB181991, accessed on 16 December 2022) was used as the internal reference gene, and the RT-PCR kit from Vazyme Company (Q341) was used. The PCR reaction system is presented in Table 5. PCR reaction conditions were 95 °C for 90 s, and 95 °C for 5 s, 60 °C for 15 s, and 72 °C for 20 s, for 40 cycles. Melting curve analysis was performed with a temperature gradient of 65–95 °C. The primers used for RT-qPCR are listed in Table 6. The data were processed according to the 2^−ΔΔCt^ method, and each sample was subjected to four technical replicates.

## 5. Conclusions

Utilizing RNA-seq technology, we conducted a comparative transcriptome analysis in this study using FHB medium-resistant and FHB medium-susceptible wheat genotypes (Shannong 102 and Nankang 1) for various periods after *Fusarium graminearum* infection. The pathways involved in the KEGG analysis of differential expression both Nankang 1 and Shannong 102 contained two highly enriched pathways, that is, the biosynthesis of secondary metabolites (ko01110) and metabolic pathways (ko01100). However, as compared to Shannong 102, Nankang 1 showed alterations in glutathione metabolism (ko00480), phenylpropanoid biosynthesis (ko00940), plant–pathogen interaction (ko04626), plant hormone signal transduction (ko04075), and other pathways, with respect to the number of upregulated genes. In this study, many PR protein genes were differentially upregulated, and these were enriched in the plant–pathogen interaction (ko04626) pathway. These genes’ expressions showed an increasing trend with the inoculation time. It shows that PR protein genes might be related to resisting invasion by *F. graminearum* and play a positive regulatory role in this process. In general, our study deepened our understanding of the complicated mechanism of FHB in wheat.

## Figures and Tables

**Table 1:** Transcriptome sequencing data analysis.

Sample	Total	Unique Mapped (%)	Multiple Mapped (%)	Total Mapped (%)	≥Q30 (%)	GC (%)
SN-0-1	53,437,598	90.57	5.02	95.58	94.28	60.01
SN-0-2	66,258,116	90.27	5.04	95.31	93.86	58.98
SN-0-3	40,958,454	90.49	4.79	95.28	93.88	58.60
SN-48-1	51,240,494	89.45	3.73	93.17	94.42	61.37
SN-48-2	48,617,354	90.54	4.10	94.64	93.84	58.50
SN-48-3	61,489,210	90.13	4.21	94.34	94.03	58.89
SN-96-1	52,242,470	79.70	3.64	83.34	94.30	59.72
SN-96-2	55,219,698	76.89	3.65	80.54	94.24	59.16
SN-96-3	58,356,204	80.23	3.84	84.07	94.24	58.85
K1-0-1	47,633,842	90.62	5.44	96.05	94.17	59.00
K1-0-2	58,276,138	91.05	5.01	96.06	94.19	59.03
K1-0-3	52,976,254	90.64	5.23	95.87	93.82	59.12
K1-48-1	89,254,412	90.04	4.94	94.98	93.66	59.46
K1-48-2	56,188,174	89.32	4.61	93.93	94.18	59.19
K1-48-3	50,330,048	90.52	5.05	95.57	94.20	59.52
K1-96-1	63,193,192	76.02	3.44	79.47	94.07	59.21
K1-96-2	51,992,614	78.60	3.58	82.18	94.30	59.25
K1-96-3	52,012,908	82.05	3.90	85.95	94.08	59.18

**Table 2:** Analysis of the significant enrichment of KEGG pathways in Shannong 102 (top 10).

Sample	Pathway ID	Pathway	Candidate Genes with Pathway Annotation
SN-48 vs. SN-0	ko01100	Metabolic pathways	2855 (49.9%)
	ko01110	Biosynthesis of secondary metabolites	1844 (32.23%)
	ko00940	Phenylpropanoid biosynthesis	436 (7.62%)
	ko04075	Plant hormone signal transduction	336 (5.87%)
	ko01200	Carbon metabolism	309 (5.4%)
	ko01230	Biosynthesis of amino acids	300 (5.24%)
	ko04626	Plant–pathogen interaction	286 (5%)
	ko00480	Glutathione metabolism	243 (4.25%)
	ko00500	Starch and sucrose metabolism	240 (4.2%)
	ko04016	MAPK signaling pathway—plant	201 (3.51%)
SN-96 vs. SN-0	ko01100	Metabolic pathways	3005 (50.23%)
	ko01110	Biosynthesis of secondary metabolites	1939 (32.41%)
	ko00940	Phenylpropanoid biosynthesis	474 (7.92%)
	ko04075	Plant hormone signal transduction	357 (5.97%)
	ko01200	Carbon metabolism	344 (5.75%)
	ko00480	Glutathione metabolism	291 (4.86%)
	ko04626	Plant–pathogen interaction	289 (4.83%)
	ko01230	Biosynthesis of amino acids	283 (4.73%)
	ko00500	Starch and sucrose metabolism	262 (4.38%)
	ko04016	MAPK signaling pathway—plant	222 (3.71%)
SN-96 vs. SN-48	ko01100	Metabolic pathways	1706 (52.54%)
	ko01110	Biosynthesis of secondary metabolites	1138 (35.05%)
	ko00940	Phenylpropanoid biosynthesis	273 (8.41%)
	ko01200	Carbon metabolism	231 (7.11%)
	ko04075	Plant hormone signal transduction	198 (6.1%)
	ko01230	Biosynthesis of amino acids	197 (6.07%)
	ko00480	Glutathione metabolism	182 (5.61%)
	ko04626	Plant–pathogen interaction	153 (4.71%)
	ko04016	MAPK signaling pathway—plant	133 (4.1%)
	ko00500	Starch and sucrose metabolism	132 (4.07%)

**Table 3:** Analysis of the significant enrichment of KEGG pathways in Nankang 1 (top 10).

Sample	Pathway ID	Pathway	Candidate Genes with Pathway Annotation
K1-48 vs. K1-0	ko01100	Metabolic pathways	2526 (45.89%)
	ko01110	Biosynthesis of secondary metabolites	1650 (29.97%)
	ko03010	Ribosome	621 (11.28%)
	ko00940	Phenylpropanoid biosynthesis	384 (6.98%)
	ko01200	Carbon metabolism	340 (6.18%)
	ko04626	Plant–pathogen interaction	321 (5.83%)
	ko01230	Biosynthesis of amino acids	314 (5.7%)
	ko04075	Plant hormone signal transduction	263 (4.78%)
	ko00480	Glutathione metabolism	227 (4.12%)
	ko04016	MAPK signaling pathway—plant	200 (3.63%)
K1-96 vs. K1-0	ko01100	Metabolic pathways	2877 (50.44%)
	ko01110	Biosynthesis of secondary metabolites	1901 (33.33%)
	ko00940	Phenylpropanoid biosynthesis	437 (7.66%)
	ko01200	Carbon metabolism	377 (6.61%)
	ko04075	Plant hormone signal transduction	358 (6.28%)
	ko01230	Biosynthesis of amino acids	299 (5.24%)
	ko04626	Plant–pathogen interaction	286 (5.01%)
	ko00480	Glutathione metabolism	267 (4.68%)
	ko00500	Starch and sucrose metabolism	252 (4.42%)
	ko04016	MAPK signaling pathway—plant	220 (3.86%)
K1-96 vs. K1-48	ko01100	Metabolic pathways	2604 (46.11%)
	ko01110	Biosynthesis of secondary metabolites	1594 (28.23%)
	ko03010	Ribosome	640 (11.33%)
	ko01200	Carbon metabolism	364 (6.45%)
	ko00940	Phenylpropanoid biosynthesis	337 (5.97%)
	ko01230	Biosynthesis of amino acids	303 (5.37%)
	ko00500	Starch and sucrose metabolism	249 (4.41%)
	ko00480	Glutathione metabolism	203 (3.59%)
	ko00010	Glycolysis/Gluconeogenesis	198 (3.51%)
	ko03013	RNA transport	188 (3.33%)

**Table 4:** KEGG classification of common differentially expressed genes (DEGs) in Shannong 102 and Nankang 1 (top 10).

Sample	Pathway ID	Pathway	Candidate Genes with Pathway Annotation
Shannong 102 vs. Nankang 1	ko01100	Metabolic pathways	281 (20.26%)
	ko01110	Biosynthesis of secondary metabolites	205 (14.78%)
	ko00480	Glutathione metabolism	81 (5.84%)
	ko00940	Phenylpropanoid biosynthesis	71 (5.12%)
	ko01200	Carbon metabolism	52 (3.75%)
	ko04075	Plant hormone signal transduction	36 (2.60%)
	ko04626	Plant–pathogen interaction	29 (2.09%)
	ko01230	Biosynthesis of amino acids	29 (2.09%)
	ko00010	Glycolysis/Gluconeogenesis	27 (1.95%)
	ko04016	MAPK signaling pathway—plant	25 (1.80%)

**Table 5:** Procedure for PCR amplification.

Composition	Volumn (μL)
2×ChamQ SYBR qPCR Master Mix	10
PCR Forward Primer (10μM)	0.4
PCR Reverse Primer (10μM)	0.4
cDNA	4
ddH_2_O	5.2
Total	20

**Table 6:** Primers used for RT-qPCR.

Gene	Forward Primer (5′-3′)	Reverse Primer (3′-5′)
TraesCS5A02G439700	TACTACGACCACGGCAGCAA	ATACAACTTCCATGAATCGCAACAC
TraesCS5B02G442900	ACGGGGAGAACCTCTACGGA	CAGATGATGAACACACCGTC
TraesCS5B02G443300	ACGGGGAGAACCTCTACGGA	CAGATGATGAACACACCGTC
TraesCS5B02G443400	ACGGGGAGAACCTCTACGGA	CAGATGATGAACACACCGTC
TraesCS5D02G446900	ACGGGGAGAACATCTACGGA	GCTCACCCCCTCGTAGTTG
ACTIN	CACTTGGTTCTCCTGCCTCT	AGGAAACTGCTGCGAGGATG

## Data Availability

All data used during the current study are included in this published article or are available from the corresponding author upon reasonable request.

## Supplementary Materials

The following supporting information can be downloaded at: https://www.mdpi.com/article/10.3390/ijms24044222/s1.

## References

[1] Ma Z.Q., Xie Q., Li G.Q., Jia H.Y., Zhou J.Y., Kong Z.X., Li N., Yuan Y. (2020). Germplasms, genetics and genomics for better control of disastrous wheat *Fusarium* head blight. Theor. Appl. Genet..

[2] Liu W.C., Liu Z.D., Huang C., Lu M.H., Liu J., Yang Q.P. (2016). Statistics and analysis of crop yield losses caused by main diseases and insect pests in recent 10 years. Plant Prot..

[3] Bai G., Shaner G. (1994). Scab of wheat: Prospects for control. Plant Dis..

[4] Agriopoulou S., Stamatelopoulou E., Varzakas T. (2020). Advances in Occurrence, Importance, and Mycotoxin Control Strategies: Prevention and Detoxification in Foods. Foods.

[5] Shi J.R., Liu X., Qiu J.B., Ji F., Xu J.H., Dong F., Yin X., Ran J. (2014). Deoxynivalenol contamination in wheat and its management. Sci. Agric. Sinica..

[6] Goswami R.S., Kistler H.C. (2004). Heading for disaster: *Fusarium graminearum* on cereal crops. Mol. Plant Pathol..

[7] McMullen M., Bergstrom G., De Wolf E., Dill-Macky R., Hershman D., Shaner G., Van Sanford D. (2012). A unified effort to fight an enemy of wheat and barley: *Fusarium* head blight. Plant Dis..

[8] Kriss A.B., Paul P.A., Xu X., Nicholson P., Doohan F.M., Hornok L., Rietini A., Edwards S.G., Madden L.V. (2012). Quantification of the relationship between the environment and *Fusarium* head blight, Fusarium pathogen density, and mycotoxins in winter wheat in Europe. Eur. J. Plant Pathol..

[9] Obanor F., Neate S., Simpfendorfer S., Sabburg R., Wilson P., Chakraborty S. (2013). *Fusarium graminearum* and *Fusarium pseudograminearum* caused the 2010 head blight epidemics in Australia. Plant Pathol..

[10] Van der Lee T., Zhang H., van Diepeningen A., Waalwijk C. (2015). Biogeography of *Fusarium graminearum* species complex and chemotypes: A review. Food Addit. Contam. Part A Chem. Anal. Control Expo. Risk Assess..

[11] Bai G., Shaner G. (2004). Management and resistance in wheat and barley to *Fusarium* head blight. Annu. Rev. Phytopathol..

[12] Zhu Z., Hao Y., Mergoum M., Bai G., Humphreys G., Cloutier S., Xia X., He Z. (2019). Breeding wheat for resistance to *Fusarium* head blight in the Global North: China, USA, and Canada. Crop J..

[13] Mesterházy A. (1995). Types and components of resistance to *Fusarium* head blight of wheat. Plant Breed..

[14] Pan Y., Liu Z., Rocheleau H., Fauteux F., Wang Y., McCartney C., Ouellet T. (2018). Transcriptome dynamics associated with resistance and susceptibility against *Fusarium* head blight in four wheat genotypes. BMC Genom..

[15] Li X., Zhong S., Chen W., Fatima S.A., Huang Q., Li Q., Tan F., Luo P. (2018). Transcriptome Analysis Identifies a 140 kb Region of Chromosome 3B Containing Genes Specific to *Fusarium* Head Blight Resistance in Wheat. Int. J. Mol. Sci..

[16] Xu J., Shi S., Wang Z., Ren H., Wang Y., Li C. (2020). Identification of FHB Resistance Genes in Wheat Cultivar Xinong 979 by Transcriptome sequencing. J. Triticeae Crop.

[17] Su P., Zhao L., Li W., Zhao J., Yan J., Ma X., Li A., Wang H., Kong L. (2021). Integrated metabolo-transcriptomics and functional characterization reveals that the wheat auxin receptor *TIR1* negatively regulates defense against *Fusarium graminearum*. J. Integr. Plant Biol..

[18] Cainong J.C., Bockus W.W., Feng Y., Chen P., Qi L., Sehgal S.K., Danilova T.V., Koo D.-H., Friebe B., Gill B.S. (2015). Chromosome engineering, mapping, and transferring of resistance to *Fusarium* head blight disease from Elymus tsukushiensis into wheat. Theor. Appl. Genet..

[19] Jia H., Zhou J., Xue S., Li G., Yan H., Ran C., Zhang Y., Shi J., Jia L., Wang X. (2017). A journey to understand wheat *Fusarium* head blight resistance in the Chinese wheat landrace Wangshuibai. Crop J..

[20] Li G., Zhou J., Jia H., Gao Z., Fan M., Luo Y., Zhao P., Xue S., Li N., Yuan Y. (2019). Mutation of a histidine-rich calcium-binding-protein gene in wheat confers resistance to *Fusarium* head blight. Nat. Genet..

[21] Qi L.L., Pumphrey M.O., Friebe B., Chen P.D., Gill B.S. (2008). Molecular cytogenetic characterization of alien introgressions with gene *Fhb3* for resistance to *Fusarium* head blight disease of wheat. Theor. Appl. Genet..

[22] Su Z., Bernardo A., Tian B., Chen H., Wang S., Ma H., Cai S., Liu D., Zhang D., Li T. (2019). A deletion mutation in TaHRC confers *Fhb1* resistance to *Fusarium* head blight in wheat. Nat. Genet..

[23] Wang H., Sun S., Ge W., Zhao L., Hou B., Wang K., Lyu Z., Chen L., Xu S., Guo J. (2020). Horizontal gene transfer of *Fhb7* from fungus underlies *Fusarium* head blight resistance in wheat. Science..

[24] Xue S., Li G., Jia H., Xu F., Lin F., Tang M., Wang Y., An X., Xu H., Zhang L. (2010). Fine mapping *Fhb4*, a major QTL conditioning resistance to *Fusarium* infection in bread wheat (*Triticum aestivum L.*). Theor. Appl. Genet..

[25] Xue S., Xu F., Tang M., Zhou Y., Li G., An X., Lin F., Xu H., Jia H., Zhang L. (2011). Precise mapping *Fhb5*, a major QTL conditioning resistance to *Fusarium* infection in bread wheat (*Triticum aestivum L.*). Theor. Appl. Genet..

[26] Xu F., Li W., Yan S., Zhang C., Zheng J., Du J. (2017). Analysis of pyramiding effect of major QTLs for resistance to scab in wheat. J. Triticeae Crop.

[27] Uauy C. (2017). Wheat genomics comes of age. Curr. Opin. Plant Biol..

[28] Martin L., Fei Z., Giovannoni J., Rose J.K.C. (2013). Catalyzing plant science research with RNA-seq. Front. Plant Sci..

[29] Jia C.L., Zhang Y., Zhu L., Zhang R. (2015). Application progress of transcriptome sequencing technology in biological sequencing. Mol. Plant Breed..

[30] Ke P., Liu Y., Zhang T., Sun Y., He L., Xiao J., Wang X. (2019). Function analysis of the key gene *TaACS2* in ethylene synthesis pathway in resistance to *Fusarium* head blight of wheat. Acta Agric. Shanghai.

[31] Schweiger W., Steiner B., Ametz C., Siegwart G., Wiesenberger G., Berthiller F., Lemmens M., Jia H., Adam G., Muehlbauer G.J. (2013). Transcriptomic characterization of two major F usarium resistance quantitative trait loci (QTL s), *Fhb1* and *Qfhs.ifa-5A*, identifies novel candidate genes. Mol. Plant Pathol..

[32] Ding L., Xu H., Yi H., Yang L., Kong Z., Zhang L., Xue S., Jia H., Ma Z. (2011). Resistance to hemi-biotrophic *F. graminearum* infection is associated with coordinated and ordered expression of diverse defense signaling pathways. PLoS ONE.

[33] Wang L., Li Q., Liu Z., Surendra A., Pan Y., Li Y., Zaharia L.I., Ouellet T., Fobert P.R. (2018). Integrated transcriptome and hormone profiling highlight the role of multiple phytohormone pathways in wheat resistance against *Fusarium* head blight. PLoS ONE.

[34] Warth B., Parich A., Bueschl C., Schoefbeck D., Neumann N.K.N., Kluger B., Schuster K., Krska R., Adam G., Lemmens M. (2015). GC-MS based targeted metabolic profiling identifies changes in the wheat metabolome following deoxynivalenol treatment. Metabolomics.

[35] Biselli C., Bagnaresi P., Faccioli P., Hu X., Balcerzak M., Mattera M.G., Yan Z., Ouellet T., Cattivelli L., Valè G. (2018). Comparative Transcriptome Profiles of Near-Isogenic Hexaploid Wheat Lines Differing for Effective Alleles at the 2DL FHB Resistance QTL. Front. Plant Sci..

[36] Sattler S.E., Funnell-Harris D.L. (2013). Modifying lignin to improve bioenergy feedstocks: Strengthening the barrier against pathogens. Front. Plant Sci..

[37] Gunnaiah R., Kushalappa A.C. (2014). Metabolomics deciphers the host resistance mechanisms in wheat cultivar Sumai-3, against trichothecene producing and non-producing isolates of *Fusarium graminearum*. Plant Physiol. Bioch..

[38] Kosaka A., Manickavelu A., Kajihara D., Nakagawa H., Ban T. (2015). Altered gene expression profiles of wheat genotypes against Fusarium head blight. Toxins.

[39] Li Y., Fu X., Zhao M., Zhang W., Li B., An D., Li J., Zhang A., Liu R., Liu X. (2018). A genome-wide view of transcriptome dynamics during early spike development in bread wheat. Sci. Rep..

[40] Pritsch C., Muehlbauer G.J., Bushnell W.R., Somers D.A., Vance C.P. (2000). Fungal development and induction of defense response genes during early infection of wheat spikes by *Fusarium graminearum*. Mol. Plant Microbe Interact..

[41] Li Z., Huang J., Wang Z., Meng F., Zhang S., Wu X., Zhang Z., Gao Z. (2019). Overexpression of Arabidopsis Nucleotide-Binding and Leucine-Rich Repeat Genes RPS2 and RPM1 (D505V) Confers Broad-Spectrum Disease Resistance in Rice. Front. Plant Sci..

[42] Chen S., Zhou Y., Chen Y., Gu J. (2018). Fastp: An ultra-fast all-in-one FASTQ preprocessor. Bioinformatics.

[43] Li H., Durbin R. (2009). Fast and accurate short read alignment with Burrows–Wheeler transform. Bioinformatics.

[44] TrapnTrapnell C., Williams B.A., Pertea G., Mortazavi A., Kwan G., Van Baren M.J., Salzberg S.L., Wold B.J., Pachter L. (2010). Transcript assembly and quantification by RNA-Seq reveals unannotated transcripts and isoform switching during cell differentiation. Nat. Biotechnol..

