# Supplementary material for: Transcriptome Dynamic Analysis Reveals New Candidate Genes Associated with Resistance to Fusarium Head Blight in Two Chinese Contrasting Wheat Genotypes
Source: Int J Mol Sci. 2023 Feb 20;24(4):4222. doi: 10.3390/ijms24044222 (PMC9966423; doi:10.3390/ijms24044222)
Supplement: Supplementary file 1 [file ijms-24-04222-s001.zip › ijms-2135499-supplementary.pdf]

Supplementary Material

1 Supplementary Figures and Tables

1.1 Supplementary Figures

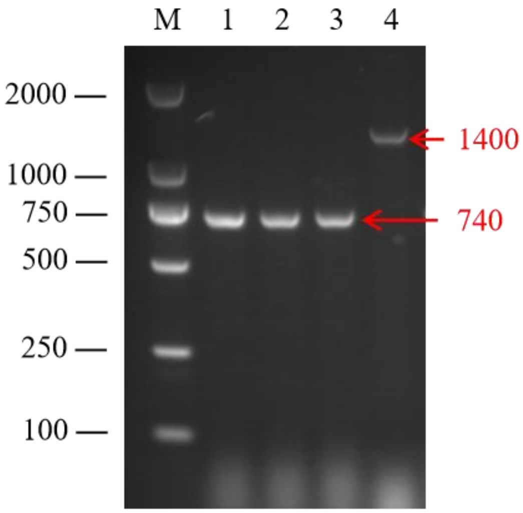

Supplementary Figure S1. Amplification results of *Fhb1* primers in the materials.

M：DL2000 DNA maker；1~4：namely Wangshuibai, Sumai 3, Nankang 1, Shannong102.

1.2 Supplementary Tables

Supplementary Table S1. The different QTL combinations in the materials.

Variety number	QTL				Number of resistance genes	Resistance[a]
	Fhb1	*Fhb2*	*Fhb4*	*Fhb5*		
Wangshuibai	+	+	+	+	4	HR
Sumai 3	+	+	-	+	3	HR
Shannong 102	-	+	+	-	2	MS
Nankang 1	+	-	+	+	3	MR

a All spikes were classified into five classes of disease severity according to the diseased spikelet rate (DSR): 0%–25% (HR), 26–50% (MR), 51–75% (MS), and 76–100% (HS).

Supplementary Table S2. Upregulated genes of GO enrichment classification (Shannong102).

	SN-48 vs SN-0			SN-96 vs SN-0			SN-96 vs SN-48		
	GO ID	GO Term	Gene Number	GO ID	GO Term	Gene Number	GO ID	GO Term	Gene Number
BP	GO:0008152	metabolic process	3496	GO:0008152	metabolic process	3585	GO:0008152	metabolic process	1697
	GO:0009987	cellular process	3211	GO:0009987	cellular process	3321	GO:0009987	cellular process	1408
	GO:0044699	single-organism process	2286	GO:0044699	single-organism process	2471	GO:0044699	single-organism process	1235
	GO:0065007	biological regulation	833	GO:0065007	biological regulation	903	GO:0065007	biological regulation	455
	GO:0050789	regulation of biological process	789	GO:0050789	regulation of biological process	832	GO:0051179	localization	428
	GO:0071840	cellular component organization or biogenesis	780	GO:0071840	cellular component organization or biogenesis	748	GO:0050789	regulation of biological process	404
	GO:0051179	localization	610	GO:0051179	localization	737	GO:0050896	response to stimulus	323
	GO:0050896	response to stimulus	584	GO:0050896	response to stimulus	628	GO:0071840	cellular component organization or biogenesis	239
	GO:0032502	developmental process	354	GO:0032502	developmental process	335	GO:0032502	developmental process	111
	GO:0032501	multicellular organismal process	274	GO:0032501	multicellular organismal process	253	GO:0023052	signaling	89
	GO:0000003	reproduction	200	GO:0000003	reproduction	180	GO:0032501	multicellular organismal process	82

	GO:0022414	reproductive process	177	GO:0023052	signaling	173	GO:0051704	multi-organism process	41
	GO:0023052	signaling	152	GO:0022414	reproductive process	155	GO:0000003	reproduction	36
	GO:0048519	negative regulation of biological process	109	GO:0048519	negative regulation of biological process	93	GO:0048518	positive regulation of biological process	36
	GO:0048518	positive regulation of biological process	60	GO:0048518	positive regulation of biological process	76	GO:0022414	reproductive process	33
	GO:0051704	multi-organism process	54	GO:0051704	multi-organism process	64	GO:0040007	growth	22
	GO:0002376	immune system process	39	GO:0040007	growth	38	GO:0048519	negative regulation of biological process	19
	GO:0040007	growth	38	GO:0002376	immune system process	30	GO:0002376	immune system process	13
	GO:0048511	rhythmic process	14	GO:0048511	rhythmic process	18	GO:0048511	rhythmic process	6
	GO:0022610	biological adhesion	13	GO:0022610	biological adhesion	10	GO:0001906	cell killing	2
	GO:0040011	locomotion	3	GO:0098754	detoxification	3	GO:0098754	detoxification	2
	GO:0098754	detoxification	2	GO:0040011	locomotion	1			
	GO:0005623	cell	2397	GO:0005623	cell	2408	GO:0005623	cell	909
CC	GO:0044464	cell part	2397	GO:0044464	cell part	2408	GO:0044464	cell part	909
	GO:0043226	organelle	1952	GO:0043226	organelle	2042	GO:0043226	organelle	770
	GO:0016020	membrane	1044	GO:0016020	membrane	1195	GO:0016020	membrane	523

	GO:0044425	membrane part	813	GO:0044425	membrane part	973	GO:0044425	membrane part	455
	GO:0044422	organelle part	808	GO:0044422	organelle part	862	GO:0044422	organelle part	318
	GO:0032991	macromolecular complex	680	GO:0032991	macromolecular complex	580	GO:0032991	macromolecular complex	99
	GO:0030054	cell junction	123	GO:0030054	cell junction	111	GO:0005576	extracellular region	43
	GO:0005576	extracellular region	102	GO:0005576	extracellular region	103	GO:0030054	cell junction	16
	GO:0031974	membrane-enclosed lumen	33	GO:0031974	membrane-enclosed lumen	21	GO:0031012	extracellular matrix	9
	GO:0019012	virion	16	GO:0031012	extracellular matrix	9	GO:0031974	membrane-enclosed lumen	6
	GO:0044423	virion part	16	GO:0044420	extracellular matrix component	9	GO:0044420	extracellular matrix component	6
	GO:0009295	nucleoid	5	GO:0019012	virion	6	GO:0044421	extracellular region part	3
	GO:0031012	extracellular matrix	4	GO:0044423	virion part	6	GO:0019012	virion	1
	GO:0044420	extracellular matrix component	4	GO:0044421	extracellular region part	2	GO:0044423	virion part	1
	GO:0044421	extracellular region part	2	GO:0009295	nucleoid	1			
	GO:0099512	supramolecular fiber	2						
	GO:0003824	catalytic activity	2889	GO:0003824	catalytic activity	3125	GO:0003824	catalytic activity	1584
MF	GO:0005488	binding	2562	GO:0005488	binding	2607	GO:0005488	binding	1070
	GO:0005215	transporter activity	198	GO:0005215	transporter activity	271	GO:0005215	transporter activity	163

GO:0005198	structural molecule activity	93	GO:0001071	nucleic acid binding transcription factor activity	83	GO:0001071	nucleic acid binding transcription factor activity	57
GO:0001071	nucleic acid binding transcription factor activity	64	GO:0005198	structural molecule activity	75	GO:0060089	molecular transducer activity	26
GO:0098772	molecular function regulator	30	GO:0098772	molecular function regulator	36	GO:0004871	signal transducer activity	20
GO:0060089	molecular transducer activity	16	GO:0060089	molecular transducer activity	29	GO:0098772	molecular function regulator	12
GO:0004871	signal transducer activity	14	GO:0004871	signal transducer activity	26	GO:0016209	antioxidant activity	10
GO:0000988	transcription factor activity, protein binding	10	GO:0000988	transcription factor activity, protein binding	14	GO:0000988	transcription factor activity, protein binding	9
GO:0016209	antioxidant activity	5	GO:0016209	antioxidant activity	10	GO:0009055	electron carrier activity	9
GO:0009055	electron carrier activity	3	GO:0009055	electron carrier activity	6	GO:0005198	structural molecule activity	8

Supplementary Table S3. Upregulated genes of GO enrichment classification (Nankang1).

	K1-48 vs K1-0			K1-96 vs K1-0			K1-48 vs K1-96		
	GO ID	GO Term	Gene Number	GO ID	GO Term	Gene Number	GO ID	GO Term	Gene Number
BP	GO:0008152	metabolic process	1858	GO:0008152	metabolic process	3415	GO:0008152	metabolic process	4044
	GO:0009987	cellular process	1585	GO:0009987	cellular process	3003	GO:0009987	cellular process	3372
	GO:0044699	single-organism process	1340	GO:0044699	single-organism process	2308	GO:0044699	single-organism process	2441
	GO:0065007	biological regulation	474	GO:0065007	biological regulation	864	GO:0065007	biological regulation	808
	GO:0051179	localization	443	GO:0050789	regulation of biological process	770	GO:0071840	cellular component organization or biogenesis	788
	GO:0050789	regulation of biological process	413	GO:0051179	localization	686	GO:0050789	regulation of biological process	750
	GO:0050896	response to stimulus	357	GO:0071840	cellular component organization or biogenesis	627	GO:0051179	localization	703
	GO:0071840	cellular component organization or biogenesis	272	GO:0050896	response to stimulus	596	GO:0050896	response to stimulus	598
	GO:0032502	developmental process	157	GO:0032502	developmental process	305	GO:0032502	developmental process	336
	GO:0032501	multicellular organismal process	117	GO:0032501	multicellular organismal process	242	GO:0032501	multicellular organismal process	275
	GO:0023052	signaling	81	GO:0023052	signaling	161	GO:0000003	reproduction	189

	GO:0048518	positive regulation of biological process	54	GO:0000003	reproduction	154	GO:0022414	reproductive process	168
	GO:0000003	reproduction	53	GO:0022414	reproductive process	137	GO:0023052	signaling	143
	GO:0022414	reproductive process	47	GO:0048518	positive regulation of biological process	76	GO:0048519	negative regulation of biological process	103
	GO:0051704	multi-organism process	46	GO:0051704	multi-organism process	66	GO:0051704	multi-organism process	59
	GO:0048519	negative regulation of biological process	20	GO:0048519	negative regulation of biological process	65	GO:0048518	positive regulation of biological process	56
	GO:0002376	immune system process	16	GO:0040007	growth	44	GO:0040007	growth	47
	GO:0040007	growth	16	GO:0002376	immune system process	22	GO:0002376	immune system process	36
	GO:0048511	rhythmic process	8	GO:0048511	rhythmic process	11	GO:0048511	rhythmic process	9
	GO:0001906	cell killing	3	GO:0022610	biological adhesion	10	GO:0022610	biological adhesion	9
	GO:0098754	detoxification	2	GO:0098754	detoxification	5	GO:0098754	detoxification	5
	GO:0022610	biological adhesion	1	GO:0001906	cell killing	3	GO:0040011	locomotion	4
				GO:0040011	locomotion	1			
	GO:0005623	cell	1092	GO:0005623	cell	2081	GO:0005623	cell	2802
CC	GO:0044464	cell part	1092	GO:0044464	cell part	2081	GO:0044464	cell part	2802
	GO:0043226	organelle	935	GO:0043226	organelle	1720	GO:0043226	organelle	1990

	GO:0016020	membrane	610	GO:0016020	membrane	1078	GO:0016020	membrane	1066
	GO:0044425	membrane part	526	GO:0044425	membrane part	913	GO:0032991	macromolecular complex	1053
	GO:0044422	organelle part	442	GO:0044422	organelle part	741	GO:0044422	organelle part	860
	GO:0032991	macromolecular complex	195	GO:0032991	macromolecular complex	474	GO:0044425	membrane part	835
	GO:0005576	extracellular region	55	GO:0005576	extracellular region	108	GO:0030054	cell junction	132
	GO:0030054	cell junction	12	GO:0030054	cell junction	60	GO:0005576	extracellular region	103
	GO:0031974	membrane-enclosed lumen	9	GO:0031974	membrane-enclosed lumen	21	GO:0031974	membrane-enclosed lumen	52
	GO:0031012	extracellular matrix	8	GO:0019012	virion	10	GO:0019012	virion	22
	GO:0044420	extracellular matrix component	5	GO:0044423	virion part	10	GO:0044423	virion part	22
	GO:0019012	virion	5	GO:0044420	extracellular matrix component	5	GO:0044420	extracellular matrix component	5
	GO:0044423	virion part	5	GO:0031012	extracellular matrix	5	GO:0031012	extracellular matrix	5
	GO:0044421	extracellular region part	3	GO:0044421	extracellular region part	1	GO:0009295	nucleoid	3
							GO:0044421	extracellular region part	2
	GO:0003824	catalytic activity	1660	GO:0003824	catalytic activity	2936	GO:0003824	catalytic activity	3110
MF	GO:0005488	binding	1146	GO:0005488	binding	2316	GO:0005488	binding	2888
	GO:0005215	transporter activity	156	GO:0005215	transporter activity	228	GO:0005215	transporter activity	214

GO:0001071	nucleic acid binding transcription factor activity	51	GO:0001071	nucleic acid binding transcription factor activity	90	GO:0005198	structural molecule activity	168
GO:0005198	structural molecule activity	23	GO:0005198	structural molecule activity	71	GO:0001071	nucleic acid binding transcription factor activity	62
GO:0060089	molecular transducer activity	20	GO:0060089	molecular transducer activity	31	GO:0098772	molecular function regulator	29
GO:0016209	antioxidant activity	14	GO:0098772	molecular function regulator	29	GO:0060089	molecular transducer activity	16
GO:0004871	signal transducer activity	14	GO:0004871	signal transducer activity	19	GO:0004871	signal transducer activity	11
GO:0000988	transcription factor activity, protein binding	11	GO:0000988	transcription factor activity, protein binding	13	GO:0016209	antioxidant activity	10
GO:0098772	molecular function regulator	10	GO:0016209	antioxidant activity	12	GO:0000988	transcription factor activity, protein binding	8
GO:0009055	electron carrier activity	7	GO:0009055	electron carrier activity	6	GO:0009055	electron carrier activity	6

Supplementary Table S4. Downregulated genes of GO enrichment classification (Shannong102).

	SN-48 vs SN-0			SN-96 vs SN-0			SN-96 vs SN-48		
	GO ID	GO Term	Gene Number	GO ID	GO Term	Gene Number	GO ID	GO Term	Gene Number
BP	GO:0008152	metabolic process	1846	GO:0008152	metabolic process	1881	GO:0008152	metabolic process	1123
	GO:0009987	cellular process	1404	GO:0009987	cellular process	1431	GO:0009987	cellular process	770
	GO:0044699	single-organism process	1186	GO:0044699	single-organism process	1156	GO:0044699	single-organism process	694
	GO:0065007	biological regulation	344	GO:0065007	biological regulation	350	GO:0050896	response to stimulus	188
	GO:0051179	localization	322	GO:0051179	localization	315	GO:0065007	biological regulation	174
	GO:0050789	regulation of biological process	306	GO:0050789	regulation of biological process	314	GO:0050789	regulation of biological process	155
	GO:0050896	response to stimulus	278	GO:0050896	response to stimulus	297	GO:0051179	localization	153
	GO:0071840	cellular component organization or biogenesis	98	GO:0071840	cellular component organization or biogenesis	93	GO:0071840	cellular component organization or biogenesis	94
	GO:0032502	developmental process	68	GO:0023052	signaling	60	GO:0032502	developmental process	59
	GO:0023052	signaling	59	GO:0032502	developmental process	59	GO:0032501	multicellular organismal process	36
	GO:0032501	multicellular organismal process	48	GO:0051704	multi-organism process	44	GO:0000003	reproduction	24

	GO:0051704	multi-organism process	45	GO:0032501	multicellular organismal process	40	GO:0023052	signaling	24
	GO:0048519	negative regulation of biological process	31	GO:0048519	negative regulation of biological process	29	GO:0022414	reproductive process	22
	GO:0002376	immune system process	25	GO:0002376	immune system process	27	GO:0040007	growth	14
	GO:0000003	reproduction	23	GO:0000003	reproduction	24	GO:0002376	immune system process	13
	GO:0022414	reproductive process	23	GO:0022414	reproductive process	23	GO:0048519	negative regulation of biological process	12
	GO:0048518	positive regulation of biological process	15	GO:0048518	positive regulation of biological process	17	GO:0051704	multi-organism process	10
	GO:0040007	growth	12	GO:0040007	growth	12	GO:0048518	positive regulation of biological process	7
	GO:0048511	rhythmic process	3	GO:0098754	detoxification	2	GO:0040011	locomotion	2
	GO:0001906	cell killing	2	GO:0001906	cell killing	1	GO:0098754	detoxification	2
	GO:0098754	detoxification	1				GO:0022610	biological adhesion	1
	GO:0005623	cell	659	GO:0005623	cell	704	GO:0005623	cell	548
	GO:0044464	cell part	659	GO:0044464	cell part	704	GO:0044464	cell part	548
CC	GO:0043226	organelle	534	GO:0043226	organelle	528	GO:0043226	organelle	373
	GO:0016020	membrane	482	GO:0016020	membrane	462	GO:0016020	membrane	232
	GO:0044425	membrane part	407	GO:0044425	membrane part	380	GO:0044425	membrane part	178

	GO:0044422	organelle part	121	GO:0044422	organelle part	97	GO:0032991	macromolecular complex	131
	GO:0005576	extracellular region	45	GO:0032991	macromolecular complex	70	GO:0044422	organelle part	92
	GO:0032991	macromolecular complex	42	GO:0005576	extracellular region	40	GO:0005576	extracellular region	32
	GO:0030054	cell junction	10	GO:0030054	cell junction	13	GO:0030054	cell junction	11
	GO:0031974	membrane-enclosed lumen	5	GO:0031974	membrane-enclosed lumen	7	GO:0031974	membrane-enclosed lumen	9
	GO:0009295	nucleoid	3	GO:0009295	nucleoid	3	GO:0019012	virion	4
	GO:0031012	extracellular matrix	3	GO:0019012	virion	3	GO:0044423	virion part	4
				GO:0044423	virion part	3	GO:0009295	nucleoid	3
							GO:0099512	supramolecular fiber	2
	GO:0003824	catalytic activity	2111	GO:0003824	catalytic activity	2079	GO:0003824	catalytic activity	1164
	GO:0005488	binding	1421	GO:0005488	binding	1539	GO:0005488	binding	899
	GO:0005215	transporter activity	164	GO:0005215	transporter activity	156	GO:0005215	transporter activity	45
MF	GO:0001071	nucleic acid binding transcription factor activity	44	GO:0001071	nucleic acid binding transcription factor activity	44	GO:0001071	nucleic acid binding transcription factor activity	16
	GO:0004871	signal transducer activity	11	GO:0016209	antioxidant activity	11	GO:0005198	structural molecule activity	15
	GO:0060089	molecular transducer activity	8	GO:0004871	signal transducer activity	10	GO:0016209	antioxidant activity	5
	GO:0016209	antioxidant activity	7	GO:0060089	molecular transducer activity	5	GO:0004871	signal transducer activity	3

GO:0005198	structural molecule activity	1	GO:0005198	structural molecule activity	2	GO:0000988	transcription factor activity, protein binding	1
GO:0009055	electron carrier activity	1				GO:0060089	molecular transducer activity	1
						GO:0098772	molecular function regulator	1

Supplementary Table S5. Downregulated genes of GO enrichment classification (Nankang1).

	K1-48 vs K1-0			K1-96 vs K1-0			K1-96 vs K1-48		
	GO ID	GO Term	Gene Number	GO ID	GO Term	Gene Number	GO ID	GO Term	Gene Number
BP	GO:0008152	metabolic process	2968	GO:0008152	metabolic process	1819	GO:0008152	metabolic process	934
	GO:0009987	cellular process	2256	GO:0009987	cellular process	1394	GO:0009987	cellular process	714
	GO:0044699	single-organism process	1650	GO:0044699	single-organism process	1132	GO:0044699	single-organism process	621
	GO:0065007	biological regulation	481	GO:0051179	localization	341	GO:0051179	localization	195
	GO:0051179	localization	470	GO:0065007	biological regulation	340	GO:0065007	biological regulation	189
	GO:0050789	regulation of biological process	447	GO:0050789	regulation of biological process	310	GO:0050789	regulation of biological process	167
	GO:0050896	response to stimulus	398	GO:0050896	response to stimulus	273	GO:0050896	response to stimulus	145
	GO:0071840	cellular component organization or biogenesis	384	GO:0071840	cellular component organization or biogenesis	94	GO:0071840	cellular component organization or biogenesis	57
	GO:0032502	developmental process	145	GO:0032502	developmental process	58	GO:0032502	developmental process	45
	GO:0032501	multicellular organismal process	110	GO:0023052	signaling	56	GO:0032501	multicellular organismal process	33
	GO:0000003	reproduction	81	GO:0032501	multicellular organismal process	45	GO:0048519	negative regulation of biological process	26

	GO:0022414	reproductive process	73	GO:0051704	multi-organism process	40	GO:0023052	signaling	22
	GO:0023052	signaling	69	GO:0000003	reproduction	31	GO:0000003	reproduction	19
	GO:0048519	negative regulation of biological process	59	GO:0048519	negative regulation of biological process	29	GO:0022414	reproductive process	17
	GO:0051704	multi-organism process	40	GO:0022414	reproductive process	28	GO:0051704	multi-organism process	13
	GO:0002376	immune system process	25	GO:0002376	immune system process	21	GO:0048518	positive regulation of biological process	6
	GO:0048518	positive regulation of biological process	21	GO:0048518	positive regulation of biological process	17	GO:0040007	growth	6
	GO:0040007	growth	17	GO:0040007	growth	9	GO:0002376	immune system process	4
	GO:0048511	rhythmic process	5	GO:0098754	detoxification	3	GO:0098754	detoxification	1
	GO:0040011	locomotion	3	GO:0001906	cell killing	1			
	GO:0098754	detoxification	3						
	GO:0022610	biological adhesion	1						
CC	GO:0005623	cell	1769	GO:0005623	cell	692	GO:0005623	cell	416
	GO:0044464	cell part	1769	GO:0044464	cell part	692	GO:0044464	cell part	416
	GO:0043226	organelle	1184	GO:0043226	organelle	539	GO:0043226	organelle	348
	GO:0016020	membrane	688	GO:0016020	membrane	463	GO:0016020	membrane	238

	GO:0032991	macromolecular complex	627	GO:0044425	membrane part	387	GO:0044425	membrane part	194
	GO:0044425	membrane part	505	GO:0044422	organelle part	88	GO:0044422	organelle part	67
	GO:0044422	organelle part	375	GO:0032991	macromolecular complex	63	GO:0005576	extracellular region	31
	GO:0030054	cell junction	97	GO:0005576	extracellular region	33	GO:0032991	macromolecular complex	28
	GO:0005576	extracellular region	53	GO:0030054	cell junction	12	GO:0030054	cell junction	7
	GO:0031974	membrane-enclosed lumen	24	GO:0031974	membrane-enclosed lumen	5	GO:0009295	nucleoid	3
	GO:0019012	virion	11	GO:0009295	nucleoid	4	GO:0031012	extracellular matrix	3
	GO:0044423	virion part	11	GO:0099512	supramolecular fiber	1	GO:0031974	membrane-enclosed lumen	2
	GO:0009295	nucleoid	5	GO:0044421	extracellular region part	1	GO:0044421	extracellular region part	1
	GO:0099512	supramolecular fiber	2	GO:0019012	virion	1			
	GO:0044420	extracellular matrix component	1	GO:0044423	virion part	1			
	GO:0031012	extracellular matrix	1						
	GO:0044421	extracellular region part	1						
	GO:0003824	catalytic activity	2643	GO:0003824	catalytic activity	2050	GO:0003824	catalytic activity	1147
MF	GO:0005488	binding	2347	GO:0005488	binding	1486	GO:0005488	binding	726
	GO:0005215	transporter activity	203	GO:0005215	transporter activity	156	GO:0005215	transporter activity	83

GO:0005198	structural molecule activity	98	GO:0001071	nucleic acid binding transcription factor activity	31	GO:0001071	nucleic acid binding transcription factor activity	18
GO:0001071	nucleic acid binding transcription factor activity	43	GO:0004871	signal transducer activity	8	GO:0009055	electron carrier activity	6
GO:0004871	signal transducer activity	12	GO:0016209	antioxidant activity	6	GO:0016209	antioxidant activity	2
GO:0098772	molecular function regulator	12	GO:0060089	molecular transducer activity	4	GO:0004871	signal transducer activity	2
GO:0016209	antioxidant activity	7	GO:0098772	molecular function regulator	3	GO:0060089	molecular transducer activity	2
GO:0000988	transcription factor activity, protein binding	4	GO:0000988	transcription factor activity, protein binding	2	GO:0098772	molecular function regulator	2
GO:0060089	molecular transducer activity	3	GO:0005198	structural molecule activity	2	GO:0005198	structural molecule activity	1

Supplementary Table S6. KEGG enrichment analysis of up- and downregulated differentially expressed genes in Shannong 102.

Sample	Up				Down			
	Pathway ID	Pathway	Gene Number	q value	Pathway ID	Pathway	Gene Number	q value
SN-48 vs SN-0	ko01100	Metabolic pathways	1105	4.40E-12	ko01100	Metabolic pathways	1749	4.33E-15
	ko01110	Biosynthesis of secondary metabolites	800	2.48E-69	ko01110	Biosynthesis of secondary metabolites	1043	5.99E-39
	ko00940	Phenylpropanoid biosynthesis	220	1.41E-26	ko00940	Phenylpropanoid biosynthesis	215	1.95E-04
	ko04626	Plant-pathogen interaction	199	3.42E-36	ko01200	Carbon metabolism	195	2.04E-03
	ko04075	Plant hormone signal transduction	180	1.60E-18	ko00500	Starch and sucrose metabolism	172	3.92E-18
	ko00480	Glutathione metabolism	179	2.46E-48	ko00230	Purine metabolism	146	3.16E-02
	ko04016	MAPK signaling pathway - plant	148	2.07E-38	ko00520	Amino sugar and nucleotide sugar metabolism	126	7.34E-08
	ko01230	Biosynthesis of amino acids	143	9.53E-10	ko03440	Homologous recombination	110	1.64E-07
	ko00360	Phenylalanine metabolism	80	4.64E-25	ko03030	DNA replication	108	7.43E-15
	ko00520	Amino sugar and nucleotide sugar metabolism	74	4.41E-04	ko00010	Glycolysis / Gluconeogenesis	108	1.54E-02
SN-96 vs SN-0	ko01100	Metabolic pathways	1074	2.40E-02	ko01100	Metabolic pathways	1931	9.61E-38
	ko01110	Biosynthesis of secondary metabolites	807	2.09E-56	ko01110	Biosynthesis of secondary metabolites	1132	8.68E-56

	ko00940	Phenylpropanoid biosynthesis	261	1.84E-39	ko01200	Carbon metabolism	247	6.55E-12
	ko00480	Glutathione metabolism	225	2.99E-76	ko00940	Phenylpropanoid biosynthesis	213	1.20E-03
	ko04626	Plant-pathogen interaction	210	2.67E-37	ko00500	Starch and sucrose metabolism	200	9.93E-29
	ko04075	Plant hormone signal transduction	164	1.67E-10	ko04075	Plant hormone signal transduction	193	4.16E-04
	ko01230	Biosynthesis of amino acids	154	1.94E-10	ko00630	Glyoxylate and dicarboxylate metabolism	123	1.76E-28
	ko04141	Protein processing in endoplasmic reticulum	140	3.06E-05	ko00010	Glycolysis / Gluconeogenesis	123	1.61E-04
	ko04016	MAPK signaling pathway - plant	139	1.06E-29	ko00520	Amino sugar and nucleotide sugar metabolism	119	1.06E-05
	ko00520	Amino sugar and nucleotide sugar metabolism	90	7.33E-07	ko01100	Metabolic pathways	1931	9.61E-38
	ko01110	Biosynthesis of secondary metabolites	529	1.32E-37	ko01100	Metabolic pathways	1015	1.42E-39
	ko00940	Phenylpropanoid biosynthesis	180	1.22E-29	ko01110	Biosynthesis of secondary metabolites	609	2.46E-45
	ko00480	Glutathione metabolism	145	1.07E-45	ko01200	Carbon metabolism	175	4.89E-24
SN-96vs SN-48	ko04141	Protein processing in endoplasmic reticulum	123	5.70E-12	ko04075	Plant hormone signal transduction	125	5.08E-09
	ko01230	Biosynthesis of amino acids	122	8.88E-14	ko00500	Starch and sucrose metabolism	103	6.83E-16
	ko04626	Plant-pathogen interaction	104	3.56E-10	ko00010	Glycolysis / Gluconeogenesis	75	2.17E-06
	ko04016	MAPK signaling pathway - plant	60	1.20E-05	ko00630	Glyoxylate and dicarboxylate metabolism	74	3.12E-21

ko00400	Phenylalanine, tyrosine and tryptophan biosynthesis	50	3.76E-18	ko04016	MAPK signaling pathway - plant	73	3.13E-08
ko00270	Cysteine and methionine metabolism	46	1.98E-03	ko00710	Carbon fixation in photosynthetic organisms	65	1.05E-14
ko00592	alpha-Linolenic acid metabolism	43	7.14E-12	ko00564	Glycerophospholipid metabolism	65	7.53E-10

Supplementary Table S7. KEGG enrichment analysis of up- and down regulated differentially expressed genes in Nankang 1.

Sample	Up				Down			
	Pathway ID	Pathway	Gene Number	q value	Pathway ID	Pathway	Gene Number	q value
K1-48 vs K1-0	ko01110	Biosynthesis of secondary metabolites	981	4.00E-23	ko01100	Metabolic pathways	1101	9.54E-37
	ko03010	Ribosome	569	1.50E-50	ko01110	Biosynthesis of secondary metabolites	669	3.25E-48
	ko00940	Phenylpropanoid biosynthesis	279	4.71E-17	ko01200	Carbon metabolism	188	1.97E-24
	ko04626	Plant-pathogen interaction	267	2.17E-34	ko04075	Plant hormone signal transduction	120	1.64E-05
	ko01230	Biosynthesis of amino acids	236	8.15E-17	ko00630	Glyoxylate and dicarboxylate metabolism	93	1.48E-31
	ko00480	Glutathione metabolism	190	2.00E-25	ko00710	Carbon fixation in photosynthetic organisms	91	2.93E-28
	ko03008	Ribosome biogenesis in eukaryotes	136	4.18E-13	ko00564	Glycerophospholipid metabolism	84	1.57E-16
	ko04016	MAPK signaling pathway - plant	123	5.09E-08	ko00500	Starch and sucrose metabolism	83	2.37E-06
	ko00520	Amino sugar and nucleotide sugar metabolism	108	1.69E-03	ko04016	MAPK signaling pathway - plant	77	1.22E-07
	ko00270	Cysteine and methionine metabolism	85	1.63E-02	ko00010	Glycolysis / Gluconeogenesis	76	5.42E-05
K1-96 vs K1-0	ko01100	Metabolic pathways	1034	1.53E-03	ko01100	Metabolic pathways	1842	3.75E-33
	ko01110	Biosynthesis of secondary metabolites	761	2.82E-53	ko01110	Biosynthesis of secondary metabolites	1139	1.67E-68

	ko00940	Phenylpropanoid biosynthesis	240	2.21E-34	ko01200	Carbon metabolism	280	2.11E-23
	ko00480	Glutathione metabolism	205	9.38E-66	ko04075	Plant hormone signal transduction	202	1.69E-06
	ko04626	Plant-pathogen interaction	205	1.58E-38	ko00940	Phenylpropanoid biosynthesis	197	1.12E-02
	ko04075	Plant hormone signal transduction	156	2.35E-10	ko00500	Starch and sucrose metabolism	179	9.65E-22
	ko01230	Biosynthesis of amino acids	147	2.35E-10	ko00010	Glycolysis / Gluconeogenesis	138	9.12E-09
	ko04016	MAPK signaling pathway - plant	128	3.19E-26	ko00564	Glycerophospholipid metabolism	118	2.96E-15
	ko04141	Protein processing in endoplasmic reticulum	117	1.14E-02	ko00630	Glyoxylate and dicarboxylate metabolism	116	1.33E-25
	ko00360	Phenylalanine metabolism	86	4.08E-29	ko00520	Amino sugar and nucleotide sugar metabolism	109	2.30E-04
K1-96 vs K1-48	ko01100	Metabolic pathways	592	1.07E-04	ko01100	Metabolic pathways	2010	3.73E-03
	ko01110	Biosynthesis of secondary metabolites	388	6.26E-20	ko01110	Biosynthesis of secondary metabolites	1204	1.34E-27
	ko00480	Glutathione metabolism	126	2.95E-43	ko03010	Ribosome	628	1.79E-39
	ko00940	Phenylpropanoid biosynthesis	104	2.20E-08	ko01200	Carbon metabolism	311	5.75E-17
	ko04075	Plant hormone signal transduction	102	7.77E-11	ko01230	Biosynthesis of amino acids	259	2.83E-12
	ko04626	Plant-pathogen interaction	92	2.55E-11	ko00230	Purine metabolism	196	4.64E-04
	ko04016	MAPK signaling pathway - plant	84	4.07E-21	ko00500	Starch and sucrose metabolism	180	1.30E-11
	ko00500	Starch and sucrose metabolism	69	1.01E-09	ko03013	RNA transport	180	2.12E-07

	ko00052	Galactose metabolism	41	4.62E-04	ko00010	Glycolysis / Gluconeogenesis	177	1.09E-11
	ko00280	Valine, leucine and isoleucine degradation	39	2.88E-17	ko00520	Amino sugar and nucleotide sugar metabolism	126	2.67E-03

Supplementary Table S8. Statistical analysis of significantly differentially expressed genes at three time points.

ID	Symbol	Description	log_2FC					
			SN-48 vs SN-0	SN-96 vs SN-0	SN-96vs SN-48	K1-48 vs K1-0	K1-96 vs K1-0	K1-96vs K1-78
TraesCS5A02G439700	PRMS	pathogenesis-related protein 1-9 [Triticum aestivum]	2.45	4.78	2.33	5.19	7.42	2.23
TraesCS5B02G442700	PRMS	pathogenesis-related protein 1-16 [Triticum aestivum]	8.61	9.63	1.02	13.04	15.82	2.78
TraesCS5B02G442900	PRMS	pathogenesis-related protein 1-6 [Triticum aestivum]	6.88	10.07	3.19	5.30	10.80	5.49
TraesCS5B02G443200	PRMS	pathogenesis-related protein 1-7 [Triticum aestivum]	5.98	9.68	3.70	12.86	16.89	4.03
TraesCS5B02G443300	PRMS	pathogenesis-related protein 1-7 [Triticum aestivum]	7.91	12.06	4.15	7.63	11.93	4.30
TraesCS5B02G443400	PRMS	pathogenesis-related protein 1-7 [Triticum aestivum]	4.42	8.86	4.44	12.39	16.62	4.22
TraesCS5B02G443500	PRMS	pathogenesis-related protein 1-7 [Triticum aestivum]	3.81	7.89	4.08	12.31	15.83	3.52
TraesCS5B02G443600	PRMS	pathogenesis-related protein 1-7 [Triticum aestivum]	4.40	8.02	3.62	13.41	17.78	4.37
TraesCS5B02G644500LC	PRMS	pathogenesis-related protein 1-7 [Triticum aestivum]	5.09	9.15	4.06	6.87	11.19	4.33
TraesCS5D02G446800	PRMS	pathogenesis-related protein 1-like [Aegilops tauschii subsp. tauschii] [Aegilops tauschii]	3.83	5.20	1.37	4.15	5.77	1.62
TraesCS5D02G447000	PRMS	pathogenesis-related protein 1-like [Aegilops tauschii subsp. tauschii] [Aegilops tauschii]	4.79	9.00	4.21	4.68	9.28	4.60

TraesCS7B02G105100	PRMS	pathogenesis-related protein 1-18 [Triticum aestivum]	5.47	7.90	2.42	5.66	7.84	2.18
TraesCSU02G226400	PRMS	pathogenesis-related protein 1-8 [Triticum aestivum]	5.55	9.15	3.61	5.14	9.56	4.42
TraesCSU02G409000LC	PRMS	pathogenesis-related protein 1-8 [Triticum aestivum]	6.15	9.69	3.54	5.93	9.93	4.00
TraesCS2B02G401300	RPS2	disease resistance protein RPS2-like [Aegilops tauschii subsp. tauschii] [Aegilops tauschii]	7.56	8.68	1.12	6.80	8.12	1.32
TraesCS2D02G380500	RPS2	disease resistance protein RPS2-like [Aegilops tauschii subsp. tauschii] [Aegilops tauschii]	6.62	7.95	1.33	5.67	6.76	1.09

Supplementary Table S9. Number of differentially expressed genes on different chromosomes.

Chromosome / Variety/Line	Chromosome																						
	1A	1B	1D	2A	2B	2D	3A	3B	3D	4A	4B	4D	5A	5B	5D	6A	6B	6D	7A	7B	7D	Un	Total
Shannong 102	218	226	235	319	309	339	297	325	349	235	205	177	298	298	326	190	208	186	228	230	270	286	5754
Nankang 1	251	282	286	384	374	367	361	360	340	285	268	224	327	350	347	216	264	249	314	293	315	384	6841
